# Application of small-angle X-ray scattering for differentiation among breast tumors

**DOI:** 10.4103/0971-6203.39420

**Published:** 2008

**Authors:** V. Changizi, A. Arab Kheradmand, M. A. Oghabian

**Affiliations:** Radiology and Radiotherapy Technology Department, Allied Medical Sciences School, Medical Sciences/University of Tehran, Tehran, Iran; 1 Meraj Award, Imam Khomeini Hospital, Medical Sciences/University of Tehran, Tehran, Iran; 2 Medical Physics Department, School of Medicine, Medical Sciences/University of Tehran, Tehran, Iran

**Keywords:** Breast tumor, coherent scattering, small-angle X-ray scattering

## Abstract

Small-angle X-ray scattering (SAXS) is an X-ray diffraction-based technique where a narrow collimated beam of X-rays is focused onto a sample and the scattered X-rays recorded by a detector. The pattern of the scattered X-rays carries information on the molecular structure of the material. As breast cancer is the most widespread cancer in women and differentiation among its tumors is important, this project compared the results of coherent X-ray scattering measurements obtained from benign and malignant breast tissues. The energy-dispersive method with a setup including X-ray tube, primary collimator, sample holder, secondary collimator and high-purity germanium (HpGe) detector was used. One hundred thirty-one breast-tissue samples, including normal, fibrocystic changes and carcinoma, were studied at the 6° scattering angle. Diffraction profiles (corrected scattered intensity versus momentum transfer) of normal, fibrocystic changes and carcinoma were obtained. These profiles showed a few peak positions for adipose (1.15 ± 0.06 nm^−1^), mixed normal (1.15 ± 0.06 nm^−1^ and 1.4 ± 0.04 nm^−1^), fibrocystic changes (1.46 ± 0.05 nm^−1^ and 1.74 ± 0.04 nm^−1^) and carcinoma (1.55 ± 0.04 nm^−1^, 1.73 ± 0.06 nm^−1^, 1.85 ± 0.05 nm^−1^). We were able to differentiate between normal, fibrocystic changes (benign) and carcinoma (malignant) breast tissues by SAXS. However, we were unable to differentiate between different types of carcinoma.

Breast cancer is the most widespread cancer in women. It is a major cause of death in middle-aged women of age 33-55 years.[1] In the last two decades, intensive research in breast imaging has taken place and has led to significant technological progress. Nevertheless, X-ray mammography is still the gold standard for screening. Mammography reduces breast cancer mortality[2] but has its limitations. This technique is nonspecific since X-ray attenuation has no direct connection with the presence of disease. As a result it features a high false-positive rate, with less than 20% of women recalled following a suspicious mammogram actually having breast cancer.[3] It also has a significant false-negative rate, with an overall sensitivity of 90%.[1] The sensitivity is further reduced in younger women who have a dense-background pattern or hormone-replacement therapy.[4]

In medical X-ray imaging, more than 50% of the interactions in tissues are coherent and incoherent scattering.[5] In particular, the coherent (small-angle) X-ray scatter peaks at those energies at which X-ray breast imaging is performed. The name ‘coherent scattering’ is given to those interactions in which radiation undergoes a change in direction without a change in wavelength. For this reason, the term ‘unmodified scattering’ is sometimes used. There are two types of coherent scattering: Thomson scattering and Rayleigh scattering. In Thomson scattering, a single electron is involved in the interaction. Rayleigh scattering results from a cooperative interaction with all the electrons of an atom. Both types of coherent scattering may be described in terms of a wave-particle interaction and are therefore sometimes called ‘classical scattering.’ Low-energy radiation encounters the electrons of an atom and sets them into vibration at the frequency of the radiation. A vibrating electron, because it is a charged particle, emits radiation. The process may be envisioned as the absorption of radiation, vibration of the atom and emission of radiation as the atom returns to its undisturbed state. This is the only type of interaction between X-rays and matter that does not cause ionization. Small-angle X-ray scattering (SAXS) is an X-ray diffraction-based technique where a narrow collimated beam of X-rays is focused onto a sample and the scattered X-rays recorded by a detector. The pattern of the scattered X-rays carries information on the molecular structure of the material. The momentum transferred (Q) to the photon causing it to be deflected through an angle θ is defined as

Q = (sin⁡θ/2)/λ

where λ is the wavelength.

Kosanetzky *et al.*[6] using SAXS presented diffraction patterns for some plastics and several biological samples. Their measurements showed that fat and bone have scattering profiles that are considerably different from water, liver and muscle. This technique was applied to bone by Speller *et al.*[7] and Royle *et al.*[8,9] Kidane *et al.*[1] and Changizi *et al.*[10] using SAXS made differentiation between cancerous and normal breast tissues. Hussein *et al.'s*[11] examinations showed that the SAXS method was able to identify the changes in bone density.

In order to evaluate the possible application of the small-angle X-ray scattering as a diagnostic tool, we compared the results of coherent X-ray scattering measurements obtained from benign and malignant breast tissues.

## Materials and Methods

**Figure 1:**
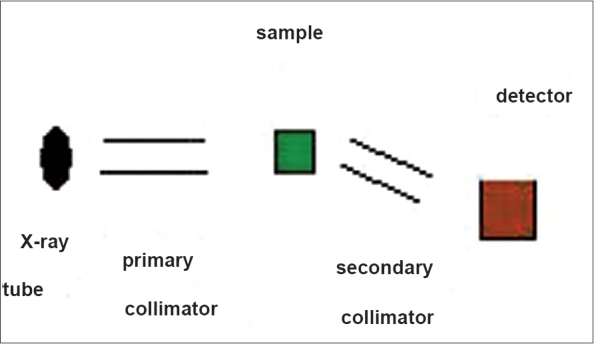
Schematic diagram of SAXS setup

The diffraction patterns can be obtained either by using monoenergetic photons and scanning the various scattering angles (angular dispersive) or by using polyenergetic photons at a certain scattering angle (energy dispersive). In this project polyenergetic photons were used, and the scattering patterns were measured at a fixed scattering angle of 6°. Figure 1 shows a schematic diagram of the small-angle X-ray scattering setup used in this study.

The tungsten target X-ray tube was operated at 10 mA and 55 kV. To obtain a pencil beam, two collimators made of lead with 50-mm length and 1-mm hole, acceptance angle approximately 1°, were used. The source-to-sample distance was 150 mm, and sample-to-detector distance was 170 mm. All system components were mounted on an isolated optical table.

The detection system was a planar HpGe detector (model GEM Ortec EG and G) with energy resolution of 450 eV at 59.7 keV. In order to minimize the background radiation, several lead rings and lead bricks were placed around the detector. The output pulses were fed to a PC-based multichannel analyzer (92X Spectrum Master, EG and G Ortec). The analyzer was used to digitize the analog information contained in each pulse, to process and store the data in memory and finally to display the contents of the processed data in the form of counts against channel number. The channel number was converted into energy of the scattered photons by performing a calibration procedure using an isotope of known energy (^241^ Am). In order to obtain the scatter signatures, all the samples with 5-mm diameter and 5-mm height were positioned at the center of the scattering volume, defined by collimation geometry. During the scatter measurements, the sample was rotated at constant speed by a step motor to ensure that all parts of the tissue sample were exposed uniformly to the beam.

The total exposure time was set to 500 s, which provided good counting statistics for the measurements. This exposure time resulted in a statistical error of 0.01 per channel.

Following corrections were done on the raw data:

1. The background counts due to the sample holder scattering were subtracted from the raw data.
2. Each measured spectrum was corrected for the shape of the incident spectrum.
3. Each measured spectrum was corrected for photons attenuation through the sample.

Finally using equation (1), energy values were transformed to momentum transfer.

One hundred thirty-one samples were collected from women who underwent mastectomy procedures at the Imam Khomeini Hospital (Tehran, Iran). A section from each sample was sent for histological analysis, whilst the remaining tissue was prepared for SAXS analysis. Following surgery, the sample was placed in a nitrogen tank and frozen at −196°C for storage. Each sample was brought into equilibrium at room temperature prior to measurement. Histological analysis of the samples gave the following breakdown of tissue types: 10 pure adipose tissues, 33 mixed normal tissues, 61 carcinoma and 27 fibrocystic changes.

In the cases of peak position and integrated intensity, Student t-test examination among different above-mentioned tissue groups with 95% confidence interval was done by SPSS program.

## Results

**Figure 2:**
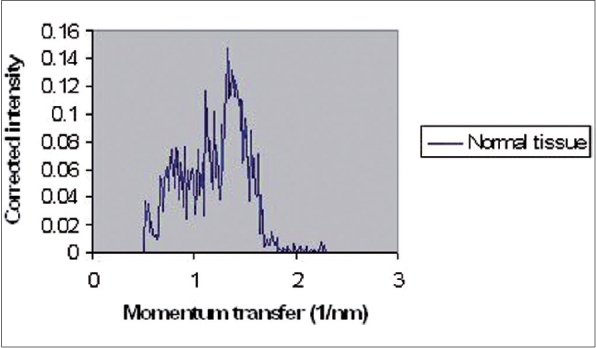
Coherent X-ray scattering of breast normal tissue (adipose and fibroglandular)

**Figure 3:**
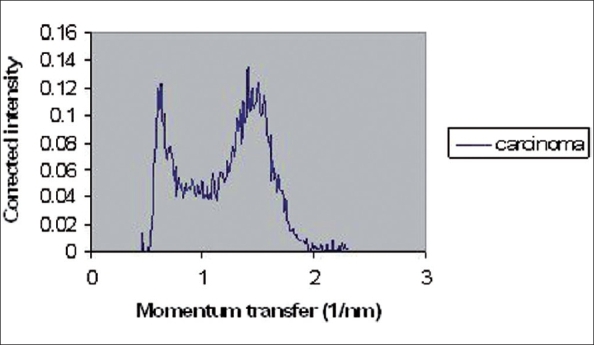
Coherent X-ray scattering of invasive carcinoma

**Figure 4:**
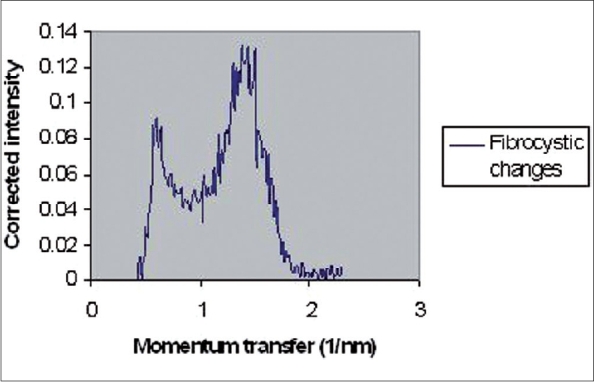
Coherent X-ray scattering of fibrocystic changes

**Figure 5:**
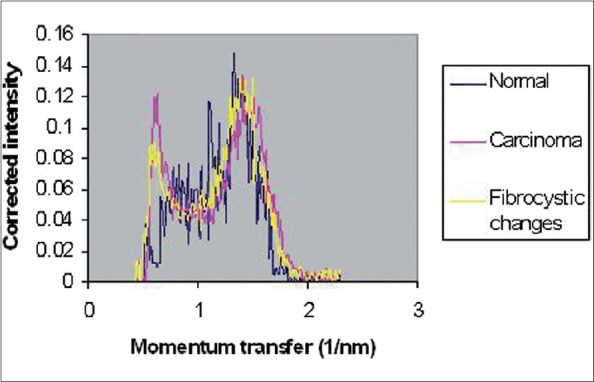
Comparison among three coherent scattering spectra of normal, carcinoma and fibrocystic changes

The coherently scattered radiation from all atoms in a material undergoes reinforcement in certain directions (constructive interference) and cancellation in other directions (destructive interference). Constructive interferences produce peaks in coherent X-ray scattering spectra. These spectra, including peaks, have been illustrated in Figures 2–5 for normal (adipose and fibroglandular), invasive carcinoma, fibrocystic changes and whole types together respectively. Peaks in each figure are related to nature of tissue. Table 1 shows certain values of peak positions obtained from different types of breast tissues. Tables 2 and 3 illustrate Student's t-test analysis regarding peak positions and coherent scattering intensity. It was found that peak position can make differentiation between normal and tumor breast tissues (*P* < 0.001). Furthermore, it is possible to differentiate between fibrocystic changes (benign tumor) and carcinoma (malignant tumor) using this parameter, even though they are very close to each other (*P* < 0.001). Different malignant breast tumors (such as ductal carcinoma, *in situ* ductal carcinoma, lobular carcinoma) showed almost the same peak positions. As a matter of fact, SASX is unable to make differentiation among them (*P* > 0.05).

**Table 1:** List of 131 breast-tissue samples classified as histological, including information related to peak position

*Tissue type*	*Number of samples*	*Peak positions (nm^−1^)*
Carcinoma	61	1.55 ± 0.04
		1.73 ± 0.06
		1.85 ± 0.05
Fibrocystic	27	1.46 ± 0.05
changes		1.74 ± 0.04
Adipose	10	1.15 ± 0.06
Adipose and	33	1.15 ± 0.06
fibroglandular		1.4 ± 0.04

**Table 2:** Significance values for the Student's t-test carried out on the peak positions associated with different types of breast disease

*Tissue type*	*Number of samples*	*Peak position(nm-1)*	*P-Value*	*95% Confidence interval of the difference*	
					
				*Lower level*	*Upper level*
Carcinoma	61	1.55 ± 0.04	*P* < 0.001	0.08	0.17
		1.73 ± 0.06			
		1.85 ± 0.05			
		1.46 ± 0.05			
Fibrocystic changes	27	1.74 ± 0.04			
Carcinoma	61	1.55 ± 0.04	*P* <0.001	0.39	0.47
		1.73 ± 0.06			
		1.85 ± 0.05			
Normal (adipose and	33	1.15 ± 0.06			
fibroglandular)		1.4 ± 0.04			
Fibrocystic changes	27	1.46 ± 0.05	*P* <0.001	0.26	0.36
		1.74 ± 0.04			
Normal (adipose and	33	1.15 ± 0.06			
fibroglandular)		1.4 ± 0.04			

**Table 3:** Significance values for the Student's t-test carried out on the normalized scattering intensity associated with different types of breast disease

*Tissue type*	*Number of samples*	*Normalized scattering intensity*	*P-Value*	*95% Confidence interval of the difference*	
					
				*Lower level*	*Upper level*
Carcinoma	61	12.1 ± 3.6	*P* > 0.05	−12.2	16.9
Fibrocystic changes	27	11.8 ± 4.9			
Carcinoma	61	12.1 ± 3.6	*P* <0.001	4.063	8.145
Normal (adipose and fibroglandular)	33	5.9 ± 0.94			
Fibrocystic changes	27	11.8 ± 4.9	*P* <0.05	1.34	10.5
Normal (adipose and fibroglandular)	33	5.9 ± 0.94			

## Discussion

Table 1 illustrates that peak positions of coherent scattering spectrum are suitable parameters for making differentiation among carcinoma, fibrocystic changes and normal breast tissues. This result is similar to that obtained by Kidane *et al.* and Changizi *et al.* Since the peak value was shown to be dependent on material characteristics, any differentiation can be mainly due to the type of sample material. Connective tissue that supports the malignant cells varies in composition from fibroblastic to densely hyaline and contains varying amounts of collagen, extracellular mucin and elastic tissue.[12] This tissue replaces the fat as the tumor invades it. So, carcinoma is typically characterized by the lack of isolated pockets of fat within its mass. Hence, it should be easy to differentiate between adipose and carcinoma. A similar situation occurs for the differentiation between adipose and fibrocystic changes. But for different malignant tumors such as ductal carcinoma, lobular carcinoma, *in situ* carcinoma, no significant difference was found. As a matter of fact, according to peak position, there is a little change among their materials; so SAXS could not identify them.

Fibrocystic changes could be separated from carcinoma by SAXS, probably since they originate from different tissues and so classified into two groups: fibrocystic changes as lobular lesions and carcinoma as glandular lesions. However, their peak positions are close to each other, and so it is difficult to separate them by looking.

Coherent scattering intensities showed no significant differences between fibrocystic changes and carcinoma. By this result, it can be concluded that there are no significant differences between their electron densities.

## Conclusion

Small-angle X-ray scattering can cause constructive interferences between coherent beams. In this study using diffraction profiles from EDXRD, a few peak positions were obtained for breast tissues, including normal [pure adipose (1.15 ± 0.06 nm^−1^) and mixed (1.15 ± 0.06 nm^−1^ and 1.4 ± 0.05 nm^−1^)], carcinoma (1.55 ± 0.04 nm^−1^, 1.73 ± 0.06 nm^−1^, 1.85 ± 0.05 nm^−1^) and fibrocystic changes (1.46 ± 0.05 nm^−1^, 1.74 ± 0.04 nm^−1^). It was found that in breast tissues, SAXS is a reliable way for differentiation between normal and tumor and also between fibrocystic changes (benign tumor) and carcinoma (*P*<0.001). However, this method is not a good technique for differentiation among types of carcinomas (*P*>0.05).

In this project, it was found that there is no significant difference for coherent scattering intensities between fibrocystic changes and carcinoma (*P*>0.05).
